# Challenges and lessons from the COVID-19 pandemic for Health Surveillance in Brazil: reflections on technologies, models, and system organization

**DOI:** 10.1590/1980-549720240049

**Published:** 2024-12-16

**Authors:** Claudio Maierovitch Pessanha Henriques, Noely Fabiana Oliveira de Moura, Priscila Bochi de Souza

**Affiliations:** I Fundação Oswaldo Cruz, Center for Epidemiology and Health Surveillance – Brasília (DF), Brazil.

**Keywords:** Unified health system, Public health surveillance, Pandemics, COVID-19

## Abstract

The term "health surveillance" encompasses a wide range of activities, including the monitoring and observation of harms and diseases. We will investigate the most well-known aspect of health surveillance, namely the monitoring of communicable diseases. To illustrate our discussion, we will use the pandemic caused by the SARS-CoV-2 virus as a case study. As the text progresses, the focus shifts to an examination of technologies, models, and the structure of the system. The most severe epidemic of our era, classified as a pandemic due to its global impact, has compelled us to reflect on a multitude of areas of knowledge. The resulting suffering should preclude any association with positive images, despite the opportunity to learn and, in some instances, observe the capacity of humans to act in solidarity. It is often observed that few things are as didactic as tragedies and mistakes. Therefore, it is worth investigating them carefully to extract the necessary feedback to correct the course of action in health. In this text, we will discuss the conditions that led health surveillance in Brazil to fail miserably in its mission in the face of the emergency triggered by the 2019 new coronavirus disease. We will also present points that deserve attention in a restructuring of the health surveillance system.

## INTRODUCTION

Health surveillance plays a crucial role in the prevention and control of harms and diseases, covering a broad scope of actions and responsibilities. In this article we focus on the surveillance of communicable diseases, with emphasis on the 2019 new coronavirus (COVID-19) pandemic, the most serious epidemic of our time. COVID-19, classified as a pandemic due to its simultaneous global spread, has brought unprecedented challenges to public health systems around the world, including Brazil.

It would not be possible to assess what happened in Brazil during the COVID-19 public health emergency without mentioning the political and institutional scenario of recent years. The Brazilian response to the pandemic confirmed what was expected with the changes in the Brazilian Unified Health System (SUS), starting in 2016, after the coup d’état that removed the then-President Dilma Rousseff and the subsequent dismantling of social policies, which worsened in the following years until 2022^1^. In 2016, the Federal Government sent to the Congress the proposal that resulted in Constitutional Amendment 95^2^, establishing a "New Tax Regime" which, among other measures, froze health sector expenses for 20 years, indexing them by the Extended National Consumer Price Index.

The depletion of health policies had significant impacts on the SUS and the health of the Brazilian population, compromising fundamental principles of the system and worsening inequalities. Budget cuts, reduced investment in public health (such as immunizations, primary health care, and disease prevention), devaluation of professionals, weakening of prevention and health promotion programs and lines of care (such as mental health, women's health, Indigenous health) were hallmarks of the period. These measures reversed achievements of the SUS over the years, disrupted the financing of Primary Health Care, and threatened the sustainability of the system.

The COVID-19 pandemic exposed weaknesses in the SUS that had been observed since 2016. In addition to the institutional throttling, the political role of the Federal Government during the crisis was even worse. The head of the Executive Branch, mirroring Donald Trump and other far-right leaders worldwide, refused to recognize the gravity of the situation, rejected protective measures, and acted as the linchpin of a political movement that continually disseminated false information, defended the spread of the virus to produce herd immunity, opposed the use of masks, and even acted to destroy the credibility of vaccines. These factors not only hampered the technical, organizational, and operational response of health surveillance, but also undermined public confidence in prevention and control measures.

In this article we investigate the conditions that led health surveillance in Brazil to fail miserably in its mission during the emergency triggered by COVID-19, analyzing the impacts of the adopted policies and the technical and institutional responses, and reflecting on possibilities for correcting the course of action in public health.

### Development

#### Health surveillance in Brazil

In the 1970s, among several institutional changes, the National Department of Rural Endemic Diseases, which had brought together, in 1956, the vertical disease control programs, was incorporated into a new structure, the Superintendence of Public Health Campaigns (*Superintendência de Campanhas de Saúde Pública* — SUCAM)^3^. It began to coexist with a new model, represented by Epidemiological Surveillance, established by law^4^ in the National Health System, which would be succeeded by the SUS.

One of the incentives for the reorganization was the resounding success achieved in the eradication of smallpox in Brazil in 1973. Other results are also worth mentioning, such as the elimination of polio, rubella, and congenital rubella syndrome, which illustrate a successful combination of practices, with emphasis on vaccination, combined with activities such as epidemiological investigation, blockade, active search, communication, and monitoring^5,6^.

The backbone of this surveillance system was conceived at a time when important transformations were still beginning. Among them, we highlight:

1. In Brazil, we have had extremely relevant political, social, and institutional changes. The same process that led to the end of the military dictatorship and the promulgation of a new Magna Carta, of a democratic and decentralizing nature, also laid the foundations for the organization of the health system. The movement towards health reform was successful in enshrining in the Constitution the principles and guidelines of the SUS, which guaranteed rights to citizens and the participation of society and gave a new role to the entities of the federation in the health area. In 1982, we once again had elections for state governors — which, in many cases, meant a reorganization of the health departments. Decentralization has led states and municipalities to gradually assume new responsibilities in structuring local health systems and their management as well as specific responsibilities related to the health of their population.
2. The globalization of the economy, with the integration of supply, consumption, and financial chains, was still an incipient process. In the 1980s and 1990s, internationalization became more relevant, with an exponential increase in the quantity and speed of transcontinental travel and the flow of goods, and the homogenization of technological, production, and consumption standards. The export of operational work, as well as traditional production parks, to low-income and poorly regulated countries meant the transfer and expansion of associated health risks. We started talking about emerging and resurgent diseases, and the triple burden of disease in our country and in many middle- and low-income countries was outlined in many colors.
3. There was the evolution and dissemination of information technologies, which were incorporated into practically all dimensions of human life. Microcomputers arrived in offices and homes, allowing leaps in the production of knowledge and in the evolution of methods of epidemiological investigation and analysis. At the end of the 20th century, connectivity became popular with the world wide web (the Internet), and in the 2010s, the use of cell phones with Internet connections (smartphones) and social networks became popular.

Such movements changed the world, Brazilian society, and the State — and, with it, the entire institutional organization and public policies. Conversely, health risks and the occurrence of public health emergencies on a global scale have increased. The concepts, methods, and tools available for epidemiological surveillance have undergone important changes, including the capacity for information sharing, research, analysis, and communication. The system has evidently changed during the half century that has passed, as we will see next. But the change in conceptual frameworks over the last 50 years has been small, especially when compared with the magnitude of the aforementioned transformations.

Surveillance of communicable diseases is not limited to the notification of diseases and harms, but is strongly represented by it. With rare exceptions, the passive model of universal notification prevails, applied both to acute diseases, which require rapid responses, and to the monitoring of long-term diseases; both to high-incidence and rare diseases. There are frequent criticisms regarding the system's low sensitivity and lack of timeliness. Furthermore, in some aspects, it is possible to observe important transitions.

Following the essence of the 1988 Constitution, the decentralization of capacities and responsibilities, which began in the 1970s, continued, giving prominence to municipalities, which became responsible for implementing health actions and local planning. This movement was particularly clear in the field of health care. Although some more complex services remained under federal and state management, the latter remained with a leading coordination role.

The same happened in epidemiological surveillance. Throughout the 1980s and then with the implementation of the SUS, many professionals were trained and practically all municipalities undertook, to some extent, epidemiological surveillance activities. However, the development of standards, planning, and a large part of the activities that require specialized teams, such as data analysis, remained the responsibility of the states and the Federal Government. At the federal level, the National Health Foundation (*Fundação Nacional de Saúde* – FNS) was created in 1990, incorporating the previously mentioned SUCAM and the Special Public Health Services Foundation. Programs previously included in various departments of the Brazilian Ministry of Health were transferred to the FNS, including the National Immunization Program (*Programa Nacional de Imunizações* – PNI). The FNS also included the National Epidemiology Center, which in 2003 had its responsibilities transferred to the Ministry of Health, when the Health Surveillance Secretariat was created.

Also at the end of the 20th century, the main computerized epidemiological information systems were developed and implemented. They have been updated, following the increased availability of microcomputers and connectivity, but, overall, the designs of these systems remain very similar to those originally conceived.

In that same period, the organization of the National Network of Public Health Laboratories, local laboratories, and regional or national reference laboratories^7^ was defined. This network, the main laboratory basis for epidemiological and health surveillance, has been continually undergoing technological updates, with emphasis on the implementation of facilities with biological safety levels and the incorporation of molecular biology techniques, with significant improvements in the capacity to diagnose infectious diseases.

The PNI was formalized in 1973, before the Epidemiological Surveillance System, and is now part of it. There were significant investments, with training of professionals, organization of immunobiological purchasing routines, construction of vaccination rooms, and installation of a robust cold chain for transport and storage. This conferred solidity and capillarity to the program, present throughout the national territory. The program has undergone incremental transformations in recent decades, among which possibly the most exuberant was the inclusion of new vaccines, with extension to all age groups. The plans to increase the national production capacity of immunobiologicals were very important, with investments in public manufacturing parks and technology transfer agreements such as those that allowed access to vaccines against COVID-19 and their local production.

Brazil followed the international movement to react to the new needs for rapid response to public health emergencies and, in 2000, event-based surveillance was introduced and a training program for professionals was organized by the Ministry of Health, called the Training Program in Epidemiology Applied to SUS Services (*Programa de Treinamento em Epidemiologia Aplicada aos Serviços do SUS* – EpiSUS). This strengthens the three levels of the SUS with professionals trained to respond to public health emergencies and, over the years, it has been expanding, with the training of field epidemiologists throughout the country.

In 2005, the Strategic Information Center for Health Surveillance Network (*Rede Centro de Informações Estratégicas em Vigilância em Saúde* – CIEVS) was implemented, a milestone in strengthening the capacity to detect, monitor, and respond to public health emergencies. As a result, the SUS began to operate in a more organized manner, in the perspective that has been incorporated into the country's health culture. Also in 2005, the first revision of the International Health Regulations (IHR) took place and, recently, driven by the COVID-19 pandemic, the World Health Assembly approved a set of amendments, aiming to improve the capacity to face emergencies.

We cannot talk about epidemiological surveillance and disease control without mentioning health care, especially primary health care. It is in these services that the community's main daily interface with the health system materializes. Primary health care is the main gateway, the one closest to where people live, and is responsible for vaccination, diagnosis, treatment, and control measures, with the support of structures of greater technological complexity. It has undergone major expansion over the last three decades, not only quantitatively, but with the implementation of the Family Health Strategy, the scope and responsibility of acting beyond the walls of health centers, in the territories where people live, work, produce and consume, exposed to the most diverse risks.

In addition to the debate on strategies, part of the history of epidemiological surveillance is the tension between the conception that its role is to obtain, work with, and disseminate information or, moreover, to act in the control of diseases and harms. In other words, is it about information and action or information for action? Legally and institutionally, this is a resolved controversy in Brazil. Both Law 8080/90^8^ and the National Policy on Health Surveillance^1^9 explain that control activities are part of the area's competence, while evidently respecting others whose responsibilities contribute to reducing or eliminating risks, such as health care, health regulation, basic sanitation, education, food, security, environmental pollution control, among many others that characterize the necessary intra- and intersectoral cooperation.

### Weaknesses of Health Surveillance during the pandemic

Thus, it was surprising and also frustrating that, despite such aspects, during the COVID-19 emergency, with rare exceptions in more organized municipalities, these definitions were apparently left aside.

Once the rapid transcontinental spread of SARS-CoV-2 was identified, there were attempts in many countries to impose barriers to delay its entry. In Brazil this also happened, especially at airports. After detecting the entry of the virus, the system was not prepared to identify local transmission. Although it was already possible to learn from the scenario in Asia, Europe, and North America, in Brazil initiatives to incorporate technology for diagnosis were slow, and even slower to disseminate it, with precarious and inopportune access.

Decisions that limited the operation of establishments, restricted the movement of people, or required the use of masks were adopted by state governments, and were often challenged by the Federal Government, which was only unable to prevent them because a decision by the Supreme Federal Court guaranteed the autonomy of federated entities enshrined in the Constitution.

National communication to the public about the epidemic figures was interrupted by the Ministry of Health, and the opportunity for information on cases, hospitalizations, and deaths only improved when extra-governmental initiatives, combined with pressure from society, were able to create alternatives. The states remained as self-coordinated sources, and the mainstream press organized a so-called consortium of vehicles that worked as an official panel, with daily updates.

Although documents were released on the definitions of suspected and confirmed cases, contact control, diagnostic testing, and control measures, particularly isolation, their application, without national coordination, had little scope, limited to municipalities that organized themselves on their own initiative. In most of the country, what was done was to count cases, hospitalizations, and deaths. Thus, the previously mentioned duality, supposedly overcome, between recommending and executing, resurfaced in practice, as a result of the Federal Government's own counteraction, and little was done to reduce the transmission of the virus, which continued its natural course.

Control measures — such as masks, restrictions on movement or gatherings of people, ventilation of environments, or testing of asymptomatic individuals — were adopted in a dispersed manner, by decisions of local or sectoral authorities or by initiatives of the society.

The provision of essential supplies for the protection of healthcare workers, laboratory tests, equipment, materials, and specific medicines was chaotic. There was a lack of oxygen in hospitals and medication for intubation and assisted ventilation.

There was no national policy to reduce the circulation and transmission of the virus^10^. Only when vaccination was adopted and began to reach significant coverage were we able to witness a decline in the daily number of hospitalizations and deaths and, after a while, after booster doses and people's greater exposure to the virus, there was a drop in the number of reported cases.

The pandemic exposed to society the existence of health surveillance, its characteristics, premises, promises, limitations, and deficiencies, particularly those related to the governance of the system and dependence on the government's political command. It brought with it the desire for something that worked better. Discussions on the model have emerged and there are even proposals on the table to create an organization inspired by the Centers for Disease Control and Prevention of the United States of America and Europe.

There is a call for improving the apparatus designed to protect people and society from health threats, whether they are sudden, such as an explosive epidemic, or insidious and persistent, such as so many endemic diseases that reduce the length and quality of life.

### Reflections to contribute to the future of surveillance

The awareness that public health emergencies have become more frequent and threatening gives more emphasis to the issue. Next, we present some aspects that we believe are fundamental for thinking about the future of surveillance in the country. Each of them would deserve a chapter to adequately support reorientations in the organization of the system.

1. Decentralized system, close to people and the territory, integrated with health care at all levels. The largest platform for health surveillance action is the primary health care network, where people and those who are sick have the most frequent contact with the SUS. Well-distributed, prepared, and equipped teams can identify diseases, adopt treatment and containment measures, and communicate them to the rest of the system. It is paramount that action also takes place outside the health centers, identifying risk situations, providing information to the population, adopting promotion and protection measures, and activating other means when their capacity for action is exceeded. Primary health care and family health teams should have public health professionals (or professionals specialized in a related area) or, at the very least, their support in nearby centers^11^. Current and future challenges do not suggest improvisation; properly trained professionals can greatly contribute to improving action in the area and generating timely information. This organization resumes the concept of health surveillance as a healthcare model^12^, with the monitoring of people's health, the identification of social, environmental, collective, and individual risks and local planning with community participation.
2. Emergency services, first aid, specialties, and hospitals cannot be left aside. These are places where the detection of serious and unusual cases is more likely and where there is greater capacity for specialized diagnosis and treatment.
3. Direct communication with the population carried out by health professionals in the region: effective communication can increase the population's adherence to programs, motivate behavioral changes, and weaken misinformation. These strategies, when well implemented, encourage disease prevention through vaccination, the promotion of healthy habits, strengthen the relationship between the population and the SUS, and increase trust in health services, which is essential for the success of public health interventions.
4. Integrated information technologies, with automatic notification based on electronic medical records and/or laboratory results. The current notification systems (Notifiable Diseases Information System [*Sistema de Informação de Agravos e Notificação*] — SINAN and the Epidemiological Surveillance Information System [*Sistema de Informação da Vigilância Epidemiológica*] — Sivep) generate rework and do not make the task easier for professionals. In addition to taking time away from healthcare activities, notification is often not carried out or is inopportune. We need a system that automatically shares data from healthcare records and laboratory results, filtered by specific algorithms, to the surveillance system, which must be in charge of cleaning the database, analyzing and adopting measures for disease control and communication. With the universalization of mobile phone connectivity, it is essential that there is also the possibility of notification via this route, increasing sensitivity and opportunity.
5. Review of surveillance and monitoring work methods and processes for each of the topics of interest included in the mandatory notification list and others deemed relevant. Each disease and harm have their own dynamics, characteristics, and meaning. However, the prevailing model of passive universal notification is currently applied to almost everyone. Work processes, technologies, and instruments must be reviewed to take into account the peculiarities and increase the coherence with the strategic objectives for each disease and its context (eradication, elimination, control, reduction of serious cases, sequelae and deaths, containment, monitoring, reduction of social and economic consequences). There are experiments with other models; for respiratory viruses, a hybrid system works, sentinel for flu-like syndrome, and universal for serious cases and deaths, with a hierarchical flow for laboratory tests. Acute flaccid paralyses are an example of a sentinel syndromic approach to detect neurotropic viruses, particularly poliovirus. For many harms and diseases, information on individual cases is not essential for detection, monitoring, and taking action. Antimicrobial resistance monitoring may have a model that is appropriate to the service or location, another logic for aggregating national data, and even a specific design for certain multidrug-resistant agents.
6. Incorporation of event-based surveillance throughout the national territory. Routine epidemiological surveillance work can be more timely and effective if combined with a system for monitoring information on possible events of interest, detected through various sources, such as:
  - news and media (reports of outbreaks or incidents published in newspapers, news websites, social media, and other media);
  - observations by health professionals (reports that may suggest unusual diseases or health conditions or changes in the pattern of occurrence);
  - community and organizational sources (information directly collected from local communities or key informants, such as community health agents, who may be aware of health events before they are formally recorded; communications from schools or professionals in education, social services, religious, sports, and other organizations that interact with the population).
  The main objective of event-based surveillance is to timely detect potential threats to public health, investigate them, and trigger immediate responses. This is especially important for controlling infectious disease outbreaks, environmental disasters, chemical, biological, radioactive emergencies, and other threats.
7. Disease surveillance based on a syndromic approach. The first information that emerges about a disease, both in the clinic and in surveillance, concerns the patients’ signs and symptoms. It may not be possible to confirm an etiological diagnosis (in the case of infectious diseases, as discussed here), but rather to characterize the manifestations as syndromes. This allows choosing the next steps for diagnosis, which often depends on laboratory tests, and to take the first initial measures quickly, without waiting for the diagnosis to be confirmed. Syndromic surveillance includes the detection and monitoring of common syndromes, such as respiratory diseases (flu-like syndrome, Severe Acute Respiratory Syndrome), gastrointestinal disorders (diarrhea, vomiting), neurological disorders (convulsions, altered consciousness, headache, mental confusion), as well as signs indicative of communicable diseases such as bleeding, jaundice, and rash. Such information may come from care provided at health centers (or from searching records and medical records, in the case of monitoring), from hospital surveillance centers, or even from emergency regulation and response centers (Mobile Emergency Service, Military Police and Fire Brigade). Ongoing evaluation of drug prescriptions and laboratory results systems may also be relevant sources. By analyzing syndromic data, surveillance can identify changes in patterns that indicate an event even before laboratory results are available. Syndromic surveillance complements traditional methods by providing warnings that can anticipate investigations and control actions.
8. Laboratory research algorithms guided by syndromic surveillance, with genetic detailing and technologies for the identification of emerging pathogens. During the emergency, we had intense work from central public health laboratories, public reference laboratories, teaching and research institutions, and private laboratories. This was the backup for confirming individual diagnoses and allowed reasonable monitoring of virus circulation. Access and coverage greatly varied and the timing was not the best. It was necessary to carry out technological updates in the midst of an emergency. In other epidemics there are always similar problems, with delays in acquiring supplies, overburden, and long response times. Surveillance requires an organized laboratory structure, with activation plans, expansion of capacity and flows that allow the rational use of resources, speed, and sensitivity for the detection of agents of epidemiological importance. For a syndromic approach to be possible, testing algorithms must follow the same logic, with increasing technological complexity that reaches the identification of pathogens through metagenomics and other sophisticated methods, so that negative final results for infectious agents are rare.
9. Access to diagnosis: laboratory, self-tests (reading and notification applications). Even though *in vitro* diagnostic technologies are organized for the surveillance logic, it is important to guarantee the right of individual access to timely and reliable diagnosis. This should include offering self-tests, in many cases, giving people greater autonomy, rapid point-of-care testing and laboratory testing. It is absolutely feasible to formulate methods for collecting information appropriate to each situation, which can greatly increase the representativeness of surveillance.
10. Vaccines for disease control and personal protection: incorporation and pathways to improve coverage. In Brazil, the existence of the National Immunization Program (PNI) — the largest public program of its kind on the planet — is a source of pride. Nevertheless, challenges persist and are renewed. Frequently mentioned as the most effective among public health sector actions, vaccination has grown in scope, with the incorporation of new vaccines, target populations, which include all age groups, geographic reach, and complexity. It is necessary to strengthen the operational base, starting with primary health care. A room where there is storage, application, guidance, recording, and management work is not enough. Likewise, the team should not be restricted to operational activities. To improve coverage and achieve the best possible results, it is important to qualify the processes in each unit or team of the Family Health Strategy, such as planning, communication, and dialogue with the community, individual guidance, monitoring coverage, identifying and searching for absentees, reducing vaccine losses, and connection with other programs, services, and institutions. This requires trained people and structure. Two fronts should be highlighted, without such mentions meaning underestimating the relevant work in progress. The incorporation of new vaccines into the official calendar is the result of several stages. The first is knowledge of the existence of the product, whether through active monitoring or through demand from experts or companies. Access to the product depends on approval by the National Health Surveillance Agency (*Agência Nacional de Vigilância Sanitária* — Anvisa) (except in situations provided for by law, of purchase by international organizations); and incorporation via the National Commission for the Incorporation of Technologies (*Comissão Nacional de Incorporação de Tecnologias* – CONITEC), based on an assessment, which involves technological characteristics, necessity, epidemiological context, suitability for the SUS, manifestation of society, and cost. During the COVID-19 emergency, the agility, competence, and transparency of Anvisa and the Ministry in these fields were impressive. This performance resulted from emergency rearrangements that cannot be sustained in a routine situation. It is paramount to resume positive aspects of that period that can become permanent. The second stage is investment in research, innovation, and development in the field of vaccines. This is not just about financing, but also organizational arrangements that can enable faster steps in both proprietary technologies and their transfer. It is important to create solid connections between producer institutions and teaching and research institutions. There is currently no clear path for someone to specialize and pursue their education in the subject; there are people who learn throughout their careers in industry and others who seek individually designed itineraries in their academic trajectories. The arrangements for the production of vaccines by Farmanguinhos (Fiocruz Institute for Drug Technology) and the Instituto Butantan [Butantan Institute] certainly shed light on the matter. Brazil has experience in policies on self-sufficiency, which must be expanded and multiplied, with emphasis on the necessary development of human capacity.
11. Innovation and production of methods for diagnosis, prevention, medicines, and other technologies. The organization of surveillance at the federal level must be professionalized to act robustly in relation to these issues, which involves:
  - prospecting the technological horizon;
  - evaluating and incorporating technologies;
  - regulatory matters, in close cooperation with Anvisa;
  - cooperation agreements;
  - financing, fundraising, investment policies, and priorities;
  - protecting intellectual property and its limits, given by public health needs;
  - creating and developing research and training programs.
  This does not mean that public laboratories should not interact, cooperate, or negotiate with organizations in other countries, as was the case with vaccines against COVID-19, but that the Federal Government must have a policy to do so and take a leading role, exercising its role in negotiation, promotion, conclusion of bi- or multilateral agreements, and diplomacy, in its broadest sense, which includes the field of international law and work with organizations. In addition to the country's direct interests, its role in solidarity between peoples and cooperation within the Global South must also be bore in mind.
12. Integration with areas dedicated to the study of climate, action on the environment (natural and built), and animal health. If, a few years ago, there was speculation about the possibility that climate change would have effects on health, today the consequences are already obvious, both due to extreme events (flooding, extreme heat, drought) and the increase in the incidence of diseases, among which vector-borne transmission is often mentioned, which expands to latitudes and altitudes where there was no transmission. About 70% of communicable diseases that have emerged in the last hundred years are zoonoses. Of the seven public health emergencies of international concern declared by the World Health Organization since 2005, six were zoonoses and the seventh, caused by an increase in polio cases in 2014, has an important environmental cause. Examples include respiratory viruses — especially influenza — and antimicrobial resistance. These are problems that can only be tackled with coordinated actions involving environmental, human, and animal health. The expression "One Health" attempts to convey the concern that such connections, known for over a hundred years, should be emphasized. There are controversies surrounding these designations, but they will not be addressed in this article.
  In practice, this means that: organizations responsible for the health of humans, animals (farm, wild, or companion animals), agricultural production, and the environment must plan and act cooperatively, with information exchange and joint planning. This is still a distant goal, as they have very different missions, institutional commitments, and forms of intervention.
  - Health surveillance must expand its spectrum of disciplines and connections, approaching entities that are still distant, such as meteorology, oceanography, ecology, environmental pollution study and control agencies, biodiversity protection, and energy. It is worth understanding how changes in the environment occur, such as the loss of biodiversity and the degradation of ecosystems, and what may influence the emergence of new pathogens and the spread of diseases.
  - It is necessary to build the capacity for integrated analysis. Avian influenza and yellow fever are examples that there can be no compartmentalization of information of epidemiological interest, especially when considering the wealth of Brazilian wildlife and the country's status as a major breeder of production animals.
13. Incorporation of knowledge developed in the field of social sciences as a subsidy for analysis, planning, investigation, and control of diseases and harms. The areas of knowledge usually involved in disease control are insufficient to face the complexity of the determination process and to adopt effective control strategies. It is important to understand the population dynamics, vulnerabilities, behaviors, relationships, culture, beliefs, knowledge, and desires. Areas that were once distant are essential for improving health surveillance — such as History, Economics, Pedagogy, Law, and Anthropology. Epidemiology, a few decades ago, adopted this approach; however, it did not happen significantly in health institutions and services. This expansion is important both for studying, researching, and understanding the dynamics of the population and diseases as well as for formulating prevention, surveillance, and response actions.
14. Joint action with social assistance, work, social security, finance, education, culture, and supply. Some diseases and harms are very common in certain segments of the population and rare in others, such as those that result from poverty and contribute to its perpetuation, called socially determined diseases, according to the current priority program of the Federal Government^2^. Many disease control programs have incorporated the practice of integrated action. The experience accumulated with AIDS and more recently with income transfer programs, already documented in several publications^13,14^, demonstrates how much can be gained in effectiveness. At the beginning of the COVID-19 pandemic, it became clear that several sectors were unprepared to act in the face of a crisis of that magnitude. The confinement of low-income people and informal workers in an emergency situation is only possible with social support. This also applies to the necessary isolation of cases and contacts, who must stay away from work and social life during the transmission period. Students can only maintain their activities remotely if there is infrastructure, planning, and resources to do so. There are numerous examples of intersectoral actions to support interventions to contain the virus, support people, and organize the functioning of the economy in exceptional situations. Planning with the participation of potentially involved actors is paramount.
15. Integrated work with health surveillance in the prevention and control of diseases. During the COVID-19 emergency, it became clear to a large part of the population that they were unaware of the role of health surveillance in the evaluation of immunobiologicals, medicines, and diagnostic methods used in health surveillance. Integration is an almost obvious need to address waterborne and foodborne diseases, healthcare-associated infections, environmental or occupational exposures to pathogens, resistance to antimicrobials and insecticides used in vector control, diseases and harms related to the use of tissues, organs or cells, with the need for rules for ventilation of collective environments and use of masks. Surveillance, taken together, can point to connections between risk situations and diseases. It is up to health surveillance organizations to formulate rules and ensure compliance, and health surveillance to monitor occurrences and the effectiveness of the rules implemented, in addition to taking action to control them.
16. Expanding the use of environmental monitoring techniques to detect diseases and harms. The practices of monitoring watercourses and waste to monitor the circulation of pathogens, such as vibrio cholerae and the poliovirus, are well-known, but not widely used. More sophisticated laboratory techniques allow for a broader spectrum, in order to detect agents early and estimate the location and intensity of circulation, which can be incorporated into routine public health practices as a rich source of information and support for tackling communicable diseases. Likewise, entomological surveillance deserves investment and the inclusion of new technologies, several of which are already available, but which have not gone beyond the limits of research institutions.
17. Articulation with society for detection, response, and social support. There are organizations that have been carrying out relevant work in the field of communicable diseases for years, especially long-term ones, such as leprosy, tuberculosis, AIDS, hepatitis, and Chagas disease. They are concerned with control, but mainly with care, treatment, and the rights of carriers. There is some theoretical formulation, isolated experiences and other ephemeral ones, under different names, such as civil, participatory, citizen, and community surveillance, among others, with broader action. The National Health Council (*Conselho Nacional de Saúde* – CNS) itself formulated a resolution in 2018 that deals with the National Policy on Health Surveillance^9^. Furthermore, the COVID-19 emergency has highlighted the leading role of civil society organizations that have acted on several fronts, including communication, testing, economic support for vulnerable people, food supply, and support for isolation. Broad initiatives, such as the *Frente pela Vida* [Front for Life], linked to the CNS, *Todos pelas Vacinas* [All for Vaccines], among many others, became very relevant in that context. Even business sectors, which usually do not have much interaction with the health sector, joined forces with public health experts and created an entity to raise funds and support the emergency response: the *Todos pela Saúde* [All for Health] initiative, which gave rise to an institute of the same name.
18. Integration of sectors and community engagement in the management of public health emergencies. Preparation and response to the pandemic must be analyzed to improve performance in other future events. The integration of surveillance with primary health care and with a sufficiently equipped laboratory network would provide timely information for the emergency context, forming a cross-cutting axis of the disaster risk management and health emergency structure^15^. Areas, such as security, education, and social assistance, should be included in the formulation of prevention, preparedness, and response plans, as well as organized civil society, whose participation is essential to promote community engagement in preventive practices and adherence to public health measures, disseminate information based on data and scientific evidence, and adjust interventions to the reality of the territory.
19. The incorporation of innovative technologies, with expansion of the capacity of information systems. More than systems dedicated to specific records, such as harms and notifiable diseases, it is increasingly necessary to work with information from large databases, both in health and other sectors (social development, social security, work, education, security, traffic, trade, production, climate, etc.). In addition to conventional techniques already in use, there are advanced tools that data science and artificial intelligence can offer for analyzing large volumes of data in real time, in order to identify patterns and trends, allowing immediate intervention. Some collaborative work has shown the potential of these technologies, such as monitoring diseases related to respiratory viruses, statistical modeling for epidemiological projections (nowcasting, forecasting), and cross-referencing disease databases with those of social programs. It is necessary to expand cooperation fronts, such as those that exist with the Center for Data and Knowledge Integration for Health (*Centro de Integração de Dados e Conhecimentos para Saúde* — CIDACS) (Fiocruz-BA), *Info-gripe* (Info-flu) and Info-dengue (Fiocruz, FGV), and the National Laboratory for Scientific Computing (*Laboratório Nacional de Computação Científica*), and to establish its own information processing capacity, with equipment, connectivity, organization, systems, and a trained team. The expansion of capacity should result in the active dissemination of timely information and easy access to organized epidemiological data for managers, health professionals, researchers, the press, and the general population, according to each need, in line with the Access to Information Law (*Lei de Acesso à Informação* – LAI) and the General Data Protection Law (*Lei Geral de Proteção de Dados Pessoais* – LGPD), avoiding unnecessary bureaucratic obstacles.
20. New institutional organization, with its own staff, stability, and career. The entire health surveillance structure at the federal level is currently contained in the Secretariat of Health and Environmental Surveillance of the Ministry of Health. As part of direct management, there are obstacles to the best performance of the area. Among them, it is worth mentioning the lack of budgetary and administrative autonomy, which makes it difficult to respond in a timely manner in situations where time is a critical factor, such as emergencies, and the fragility of the staff. There are few permanent civil servants in the Ministry of Health who have the necessary training and experience. This compromises the continuity of work and makes the necessary continuous improvement on highly complex topics unfeasible.
21. In addition to these aspects, it is worth looking at health surveillance as a State activity, and not a specific government activity. Although there may be circumstantial or political priorities, which correspond to government programs, it is an ongoing activity, the proper functioning of which has an impact on the country's health security and, therefore, on the reduction of preventable diseases and deaths. Therefore, it is desirable that the management of the area be protected by mandate, with stability to ensure that there are no interruptions in actions and programs when there are government transitions or political disputes. We cannot once again accept the complete absence of national coordination, as it happened during such a critical period as that experienced between 2020 and 2022, when the Ministry of Health was sailing aimlessly, at the mercy of speeches and decisions by the government leadership that denied scientific knowledge and the gravity of the situation.

As stated in the Federal Constitution and infra-constitutional legislation, governance is shared in a federative manner between the Federal Government, states (represented by its entity, the National Council of Health Secretaries [*Conselho Nacional de Secretários de Saúde*] — CONASS) and municipalities (represented by the National Council of Municipal Health Secretaries [*Conselho Nacional de Secretarias Municipais de Saúde*] — CONASEMS), with defined entities for discussion, negotiation, and decision.

Moreover, it is important to value social participation, via the CNS, which even has a specific committee for the topic, and the scientific community, through dedicated technical committees.

Health surveillance in Brazil was organized in an integrated manner as a system half a century ago and became part of the SUS when it was created by the 1988 Constitution, spreading across all Brazilian states and municipalities. During this period, we have made great progress, with organized systems and routines of action, obtaining significant results in the elimination and control of various diseases. The implemented model underwent adaptations to the new institutional context after the promulgation of the Federal Constitution, organizational changes and technological modernization, while maintaining its original conceptual foundations and operating logic.

This modernization was not enough to guarantee effective health surveillance, capable of dealing with an epidemic like COVID-19, in a period of great political adversity and institutional fragility.

The challenges faced during the COVID-19 pandemic included the need to readapt surveillance models, given the inability to take energetic action at a time of global public health emergency. Unfortunately, the current health surveillance and assistance system has not proven to be effective for early detection and response.

Health surveillance must be resilient and adjustable, not only to react, detect and respond, but also to anticipate health crises, being able to adapt and recover in the face of unforeseen and ongoing challenges.

The experience showed how essential a flexible, integrated, and articulated health system with government sectors and different actors in society is for a coordinated and effective response.

One of the weakest points was the lack of equity in health surveillance actions, with insufficient actions to protect different segments, such as low-income communities, minority groups, residents of rural areas or those who have less access to resources, information, and care in the prevention and control of diseases, with unequal access to technologies and the lack of digital literacy as aggravating factors.

The Brazilian experience with COVID-19 offered valuable lessons for building a health system better prepared for present and future challenges and, above all, for promoting equity. This article contributes with reflections for the future of health surveillance, which urgently needs, as an essential public policy, to revisit and restructure concepts, approaches, technologies, and its institutional organization, with emphasis on fundamental aspects. We have mentioned some of these aspects here, but each deserves a specific chapter to support system reorientations and guide the formulation of policies and practices.

## Footnotes

1 In the trajectory of the SUS, the nomenclature adopted by the National Health Council and the Ministry of Health began to consider the institutional organization of Epidemiological Surveillance as Health Surveillance.

2 Established by Decree No. 11,908, of February 6, 2024, the *Programa Brasil Saudável: Unir para Cuidar* [Healthy Brazil Program: unite to care] is an offshoot of the actions of the Interministerial Committee for the Elimination of Tuberculosis and Other Socially Determined Diseases (*Comitê Interministerial para a Eliminação da Tuberculose e de Outras Doenças Determinadas Socialmente*). The following are mentioned by name: tuberculosis, Chagas disease, schistosomiasis, lymphatic filariasis, geohelminthiasis, malaria, onchocerciasis, trachoma, HIV/AIDS, viral hepatitis, leprosy, and HTLV.
