# Supplementary material for: Challenges and lessons from the COVID-19 pandemic for Health Surveillance in Brazil: reflections on technologies, models, and system organization
Source: Rev Bras Epidemiol. 2024 Dec 16;27:e240049. doi: 10.1590/1980-549720240049 (PMC11654638; doi:10.1590/1980-549720240049)
Supplement: Translation

# Translation: Desafios e lições da pandemia de COVID-19 para a Vigilância em Saúde no Brasil: reflexões sobre tecnologias, modelos e organização do sistema

**DOI:** 10.1590/1980-549720240049.2

**Authors:** Claudio Maierovitch Pessanha Henriques, Noely Fabiana Oliveira de Moura, Priscila Bochi de Souza

**Affiliations:** I Fundação Oswaldo Cruz, Núcleo de Epidemiologia e Vigilância em Saúde – Brasília (DF), Brasil.

**Keywords:** Sistema único de saúde, Vigilância em saúde pública, Pandemias, COVID-19

## Abstract

A vigilância em saúde, como vigilância de agravos e doenças, tem um escopo bastante amplo. Discutimos aqui a face mais conhecida, que é a vigilância de doenças transmissíveis, e assumimos como mote a pandemia causada pelo vírus SARS-CoV-2. No desenvolvimento do texto, a abordagem se amplia para uma reflexão sobre tecnologias, modelo e organização do sistema. A mais grave epidemia de nossa época, classificada como pandemia por atingir simultaneamente todo o planeta, tem obrigado a reflexões que envolvem múltiplos campos do conhecimento. O sofrimento decorrente deve inibir a tentação de associá-la a qualquer imagem positiva, ainda que tenha havido oportunidade para aprender e, em algumas circunstâncias, testemunhar a capacidade humana de agir solidariamente. Poucas coisas são tão didáticas como tragédias e erros. Cabe explorá-los cuidadosamente e extrair a necessária retroalimentação para corrigir rumos da atuação em saúde. Neste texto, tratamos das condições que levaram a vigilância em saúde no Brasil a falhar fragorosamente em sua missão perante a emergência desencadeada pela doença do novo coronavírus 2019 e levantamos pontos que merecem atenção em uma reestruturação do sistema de vigilância em saúde.

## INTRODUÇÃO

A vigilância em saúde desempenha um papel crucial na prevenção e controle de agravos e doenças, abrangendo um escopo amplo de ações e responsabilidades. Este artigo enfoca a vigilância de doenças transmissíveis, com destaque para a pandemia do novo coronavírus 2019 (COVID-19), a mais grave epidemia da nossa época. A COVID-19, classificada como pandemia por sua disseminação global simultânea, trouxe à tona desafios sem precedentes para os sistemas de saúde pública em todo o mundo, incluindo o Brasil.

Não seria possível avaliar o que aconteceu no Brasil durante o período de emergência de saúde pública pela COVID-19 sem mencionar o cenário político e institucional dos anos recentes. A resposta brasileira à pandemia confirmou o que se esperava com as mudanças no Sistema Único de Saúde (SUS), a partir de 2016, após o golpe de Estado que destituiu a então presidenta Dilma Rousseff e o subsequente desmonte de políticas sociais, que se agravou nos anos seguintes, até 2022^1^. Já em 2016, o Governo Federal enviou ao Congresso a proposta que resultou na Emenda Constitucional 95^2^, instituindo "Novo Regime Fiscal", que, entre outras medidas, congelava por 20 anos as despesas do setor saúde, indexando-as pelo Índice Nacional de Preços ao Consumidor Amplo.

A desidratação das políticas de saúde teve impactos significativos no SUS e na saúde da população brasileira, comprometendo princípios fundamentais do sistema e agravando desigualdades. Cortes orçamentários, redução de investimentos em saúde pública (como imunizações, atenção primária e prevenção de doenças), desvalorização de profissionais, enfraquecimento de programas de prevenção e promoção da saúde e linhas de cuidado (como saúde mental, saúde da mulher, saúde indígena) foram marcas do período. Essas medidas reverteram conquistas do SUS ao longo dos anos, foram desestruturantes no financiamento da Atenção Primária à Saúde e ameaçaram a sustentabilidade do sistema.

A pandemia de COVID-19 expôs fragilidades do SUS que eram observadas desde 2016. Além do estrangulamento institucional, ainda pior foi o papel político do Governo Federal durante a crise. O chefe do Poder Executivo, espelhando Donald Trump e outras lideranças extremo-direitistas mundiais, negou-se a reconhecer a gravidade da situação, rejeitou as medidas de proteção e agiu como pivô de um movimento político que disseminou continuamente informações falsas, defendeu a disseminação do vírus para produzir imunidade "de rebanho", opôs-se ao uso de máscaras e até mesmo agiu para destruir a credibilidade das vacinas. Esses fatores não só prejudicaram a resposta técnica, organizacional e operacional da vigilância em saúde, mas também minaram a confiança pública nas medidas de prevenção e controle.

Este artigo explora as condições que levaram a vigilância em saúde no Brasil a falhar fragorosamente em sua missão durante a emergência desencadeada pela COVID-19, analisando os impactos das políticas adotadas e as respostas técnicas e institucionais e refletindo sobre possibilidades para corrigir os rumos da atuação em saúde pública.

### Desenvolvimento

#### A vigilância em saúde no Brasil

Na década de 1970, inaugurando diversas mudanças, o Departamento Nacional de Endemias Rurais, que reunira, em 1956, os programas verticais de controle de doenças, foi incorporado a uma nova estrutura, a Superintendência de Campanhas de Saúde Pública — SUCAM^3^. Passou a coexistir com um novo modelo, representado pela Vigilância Epidemiológica, instituída por lei^4^ no Sistema Nacional de Saúde, que seria sucedido pelo SUS.

Um dos estímulos para a reorganização foi o sucesso retumbante obtido na erradicação da varíola no Brasil, em 1973. Outros resultados também são dignos de menção, como a eliminação da poliomielite, da rubéola e da síndrome da rubéola congênita, que ilustram uma combinação bem-sucedida de práticas, com destaque para a vacinação, aliada a atividades como investigação epidemiológica, bloqueio, busca ativa, comunicação e monitoramento^5,6^.

A espinha dorsal desse sistema de vigilância foi concebida num tempo em que importantes transformações ainda estavam no início. Destacamos:

1. No Brasil, tivemos mudanças políticas, sociais e institucionais extremamente relevantes. O mesmo processo que levou ao fim da ditadura militar e à promulgação de uma nova Carta Magna, de caráter democrático e descentralizador, também trouxe as bases para a organização do sistema de saúde. O movimento pela reforma sanitária teve êxito ao consagrar na Constituição os princípios e as diretrizes do SUS que garantiu direitos aos cidadãos e a participação da sociedade e deu novo papel aos entes da federação na área de saúde. Em 1982 voltamos a ter eleições para governadores dos estados, o que, em muitos casos, significou uma reorganização das secretarias de saúde. A descentralização fez com que estados e municípios assumissem, gradativamente, novas responsabilidades na estruturação dos sistemas locais de saúde e na sua gestão, bem como atribuições específicas relativas à saúde de sua população.
2. A globalização da economia, com integração das cadeias produtivas, de consumo e financeiras, ainda era um processo incipiente. Nas décadas de 1980 e 1990 a internacionalização se tornaria mais relevante, com aumento exponencial da quantidade e da velocidade das viagens transcontinentais e dos fluxos de mercadorias, homogeneização dos padrões tecnológicos, de produção e de consumo. A exportação de trabalho operacional, bem como de parques produtivos tradicionais para países de baixa renda e pouca regulação, significou a transferência e ampliação de riscos à saúde relacionados. Passamos a falar em doenças emergentes e ressurgentes, e desenhou-se com muitas cores a tripla carga de doenças em nosso país e em boa parte daqueles de média e baixa renda.
3. Houve a evolução e a disseminação de tecnologias de informação, que foram incorporadas a praticamente todas as dimensões da vida humana. Microcomputadores chegaram aos escritórios e residências, permitindo saltos na produção de conhecimento e na evolução dos métodos de investigação e análise epidemiológica. No final do século XX foi popularizada a conectividade, com a rede mundial de computadores (*world wide web*, a internet), e na década de 2010 se popularizou o uso de telefones celulares com conexão à internet ("*smartphones*") e às redes sociais.

Tais movimentos mudaram o mundo, a sociedade brasileira e o Estado e, com ele, toda a organização institucional e as políticas públicas. Em outro plano, amplificaram-se os riscos sanitários e a ocorrência de emergências de saúde pública em dimensão mundial. Passaram por importantes mudanças os conceitos, métodos e as ferramentas à disposição da vigilância epidemiológica, incluindo a capacidade de transmissão de informações, investigação, análise e comunicação. Evidentemente o sistema mudou durante o meio século que transcorreu, como veremos a seguir. Mas a mudança dos marcos conceituais ao longo dos últimos 50 anos foi pequena, especialmente quando confrontada com a magnitude das transformações mencionadas.

A vigilância de doenças transmissíveis não se resume à notificação de doenças e agravos, mas é fortemente representada por ela. Com raras exceções, prevalece o modelo passivo, de notificação universal, aplicado tanto a doenças agudas, que exigem respostas rápidas, como ao monitoramento de doenças de longo curso; tanto a doenças de alta incidência como às raras. São frequentes críticas relativas à baixa sensibilidade e à falta de oportunidade do sistema. Outrossim, em alguns aspectos é possível constatar transições importantes.

Seguindo a essência da Constituição de 1988, a descentralização de capacidades e responsabilidades, iniciada na década de 1970, foi adiante, dando destaque aos municípios, que passaram a ser responsáveis pela execução das ações de saúde e planejamento local. Esse movimento foi particularmente claro na área de atenção à saúde. Ainda que alguns serviços mais complexos tenham permanecido sob gestão federal e dos estados, estes últimos ficaram com um papel preponderante de coordenação.

O mesmo aconteceu na vigilância epidemiológica. Ao longo da década de 1980 e, em seguida, com a implantação do SUS, muitos profissionais foram capacitados e praticamente todos os municípios assumiram, em algum grau, atividades de vigilância epidemiológica. No entanto, a elaboração de normas, o planejamento e boa parte das atividades que exigem equipes especializadas, como análise de dados, permaneceram sob responsabilidade dos estados e da União. Na esfera federal, foi criada a Fundação Nacional de Saúde (FNS) em 1990, incorporando a SUCAM, mencionada anteriormente, e a Fundação Serviços Especiais de Saúde Pública. Programas antes abrigados em várias secretarias do Ministério da Saúde foram transferidos para a FNS, inclusive o Programa Nacional de Imunizações (PNI). A FNS também abrigou o Centro Nacional de Epidemiologia, que em 2003 teve suas atribuições transferidas para o Ministério da Saúde, quando foi criada a Secretaria de Vigilância em Saúde.

Também no fim do século XX foram desenvolvidos e implantados os principais sistemas informatizados de informações epidemiológicas. Eles tiveram atualizações, acompanhando o aumento da disponibilidade de microcomputadores e conectividade, mas, em linhas gerais, os desenhos desses sistemas mantêm-se muito semelhantes aos que foram concebidos originalmente.

Naquele mesmo período foi definida a organização da Rede Nacional de Laboratórios de Saúde Pública, de laboratórios locais e de referência regional ou nacional^7^. Essa rede, principal base laboratorial das vigilâncias epidemiológica e sanitária, vem passando continuamente por atualização tecnológica, merecendo destaque a implantação de instalações com níveis de segurança biológica e a incorporação de técnicas de biologia molecular, com importante melhoria na capacidade de diagnóstico de doenças infecciosas.

O PNI foi formalizado em 1973, antes do Sistema de Vigilância Epidemiológica, e é hoje parte dele. Houve importantes investimentos, com formação de profissionais, organização de rotinas de compra de imunobiológicos, construção de salas de vacinas e instalação de uma robusta cadeia de frio para transporte e armazenamento. Isso deu solidez e capilaridade ao programa, presente em todo o território nacional. O programa passou por transformações incrementais nas últimas décadas, entre as quais possivelmente as mais exuberantes foram as de inclusão de novas vacinas, com extensão para todas as faixas etárias. Foram muito importantes os planos para aumentar a capacidade de produção nacional de imunobiológicos, com investimentos nos parques fabris públicos e acordos de transferência de tecnologia, a exemplo daqueles que permitiram o acesso a vacinas contra a COVID-19 e a produção local delas.

O Brasil acompanhou o movimento internacional de reação às novas necessidades de resposta rápida às emergências de saúde pública e, em 2000, foi introduzida a vigilância baseada em eventos e foi organizado pelo Ministério da Saúde um programa de formação de profissionais, denominado Programa de Treinamento em Epidemiologia Aplicada aos Serviços do SUS (EpiSUS). Este fortalece os três níveis do SUS com profissionais capacitados para respostas às emergências de saúde pública e, ao longo dos anos, vem se expandindo, com a formação de epidemiologistas de campo por todo o país.

Em 2005 foi implantada a Rede Centro de Informações Estratégicas em Vigilância em Saúde (CIEVS), um marco no fortalecimento da capacidade de detecção, monitoramento e resposta às emergências em saúde pública. Com isso, o SUS passou a atuar de forma mais organizada, na perspectiva que vem sendo incorporada à cultura sanitária do país. Também em 2005 ocorreu a primeira revisão do Regulamento Sanitário Internacional (RSI), e recentemente, movida pela pandemia de COVID-19, a Assembleia Mundial da Saúde aprovou um conjunto de emendas, visando aperfeiçoar a capacidade de enfrentar emergências.

É impossível falar da vigilância epidemiológica e do controle de doenças sem mencionar a atenção à saúde, especialmente a atenção primária. É nesses serviços que se materializa a principal interface cotidiana da comunidade com o sistema de saúde. A atenção primária é a principal porta de entrada, aquela mais próxima de onde vivem as pessoas, e é responsável por vacinação, diagnóstico, tratamento e medidas de controle, com a retaguarda de estruturas de maior complexidade tecnológica. Passou por grande expansão nas últimas três décadas, não apenas quantitativa, mas com a implantação da Estratégia Saúde da Família, do escopo e da responsabilidade de atuação para além dos muros das unidades de saúde, nos territórios onde vivem, trabalham, produzem e consomem as pessoas, expostas a riscos os mais diversos.

Além do debate sobre estratégias, faz parte da história da vigilância epidemiológica o tensionamento entre a concepção de que seu papel é obter, trabalhar e disseminar informações ou, além disso, atuar no controle de doenças e agravos. Ou seja, informação e ação ou informação para a ação? Legal e institucionalmente, esta é uma polêmica resolvida no Brasil. Tanto a Lei 8080/90^8^ como a Política Nacional de Vigilância em Saúde^1^9 explicitam que atividades de controle são parte da competência da área, respeitadas, evidentemente, outras cujas responsabilidades concorrem para reduzir ou eliminar riscos, como assistência à saúde, regulação sanitária, saneamento básico, educação, alimentação, segurança, controle de poluição ambiental, entre tantas que caracterizam a necessária cooperação intra e intersetorial.

### Fragilidades da Vigilância em Saúde durante a pandemia

Assim, foi surpreendente e também frustrante que, apesar de tais enunciados, durante a emergência por COVID-19, com raras exceções de municípios mais organizados, essas definições tenham aparentemente sido deixadas de lado.

Identificada a disseminação transcontinental rápida do SARS-CoV-2, houve, em muitos países, tentativas de imposição de barreiras que retardassem sua entrada. No Brasil isso também aconteceu, especialmente nos aeroportos. Depois da detecção de entrada do vírus, o sistema não estava preparado para identificar transmissão local. Embora já fosse possível aprender com o cenário na Ásia, na Europa e na América do Norte, no Brasil foram lentas as iniciativas para incorporar tecnologia para diagnóstico, mais ainda para disseminá-la, com acesso precário e inoportuno.

Decisões que limitavam o funcionamento de estabelecimentos, restringiam a circulação de pessoas ou obrigavam o uso de máscaras foram adotadas por governos estaduais, sendo muitas vezes contestadas pelo Governo Federal, que só não pôde impedi-las porque uma decisão do Supremo Tribunal Federal garantiu a autonomia dos entes federados inscrita na Constituição.

A comunicação nacional para o público sobre os números da epidemia foi interrompida pelo Ministério da Saúde, e a oportunidade de informação sobre casos, internações e óbitos só melhorou quando iniciativas extragovernamentais, aliadas à pressão da sociedade, foram capazes de criar alternativas. Os estados mantiveram-se como fontes autocoordenadas, e a grande imprensa organizou um chamado consórcio de veículos que funcionou como painel oficial, com atualização diária.

Embora tenham sido divulgados documentos sobre as definições de casos suspeitos e confirmados, controle de contatos, testes para diagnóstico e medidas de controle, particularmente o isolamento, sua aplicação, sem coordenação nacional, teve alcance pequeno, limitado a municípios que se organizaram por iniciativa própria. Em boa parte do país, o que se fez foi contabilidade de casos, internações e óbitos. Assim, aquela dualidade mencionada acima, supostamente superada, entre recomendar e executar, ressurgiu na prática, como fruto da própria contra-ação do Governo Federal, e pouco se fez para reduzir a transmissão do vírus, que continuou seu curso natural.

Medidas de controle como máscaras, restrição de circulação ou aglomeração de pessoas, ventilação de ambientes ou testagem de assintomáticos foram adotadas de forma pulverizada, por decisões de autoridades locais, setoriais ou por iniciativas da sociedade.

Foi caótico o suprimento de insumos indispensáveis para a proteção dos trabalhadores da saúde, exames laboratoriais, equipamentos, materiais e medicamentos específicos. Faltou oxigênio em hospitais e medicamentos para intubação e ventilação assistida.

Não houve política nacional para reduzir a circulação e a transmissão do vírus^10^. Somente quando a vacinação foi adotada e começou a atingir coberturas significativas pudemos assistir ao declínio do número diário de internações e óbitos e, decorrido algum tempo, após doses de reforço e grande exposição das pessoas aos vírus, houve queda na quantidade de casos notificados.

A pandemia expôs à sociedade a existência da vigilância em saúde, suas características, premissas, promessas, limitações e deficiências, particularmente as relativas à governança do sistema e dependência do comando político do governo. Trouxe junto o desejo de alguma coisa que funcionasse melhor. Emergiram discussões sobre o modelo e estão em pauta até mesmo propostas de criação de uma organização inspirada nos Centros de Controle e Prevenção de Doenças dos Estados Unidos e da Europa.

Há clamor pelo aperfeiçoamento do aparato destinado a proteger as pessoas e a sociedade de ameaças à saúde, sejam elas abruptas, como uma epidemia explosiva, ou insidiosas e permanecentes, como tantas doenças endêmicas que reduzem a duração e a qualidade de vida.

### Reflexões para contribuir com o futuro da vigilância

A consciência de que emergências de saúde pública têm se tornado mais frequentes e ameaçadoras dão mais ênfase ao tema. Apresentamos a seguir alguns aspectos que nos parecem fundamentais para pensar o futuro da vigilância no país. Cada um deles mereceria um capítulo para subsidiar adequadamente reorientações na organização do sistema.

1. Sistema descentralizado, próximo das pessoas e do território, integrado com a atenção em todos os níveis. A maior plataforma para a ação de vigilância em saúde é a rede de atenção primária à saúde, onde acontece o contato mais frequente das pessoas e de quem está doente com o SUS. Equipes bem distribuídas, preparadas e equipadas podem identificar doenças, adotar medidas de tratamento e contenção e comunicar ao restante do sistema. É fundamental que a atuação também aconteça fora das unidades, identificando situações de risco, levando informações à população, adotando medidas de promoção e proteção e acionando outros meios quando sua capacidade de ação for superada. As equipes de atenção básica e saúde da família deveriam contar com sanitaristas (ou profissionais especializados em área afim) ou, no mínimo, com a retaguarda deles em núcleos próximos^11^. Os desafios atuais e futuros contraindicam improvisações; profissionais devidamente formados podem contribuir muito para melhorar a ação no território e gerar informações oportunas. Este desenho resgata o conceito de vigilância da saúde como modelo de atenção^12^, com o monitoramento da saúde das pessoas, a identificação de riscos sociais, ambientais, coletivos e individuais e o planejamento local com participação da comunidade.
2. Serviços de urgência, pronto atendimento, especialidades e hospitais não podem ficar à margem. São locais onde é mais provável a detecção de casos graves e inusitados e onde há maior capacidade para diagnóstico e tratamento especializados.
3. Comunicação direta com a população realizada por profissionais de saúde do território: uma comunicação eficaz pode aumentar a adesão da população aos programas, motivar mudanças de comportamentos e enfraquecer a desinformação. Essas estratégias, quando bem implementadas, estimulam a prevenção de doenças por vacinação, a promoção de hábitos saudáveis, fortalecem a relação população-SUS e aumentam a confiança nos serviços de saúde, essencial para o sucesso de intervenções de saúde pública.
4. Tecnologias de informação integradas, com notificação automática baseada no registro em prontuário e/ou do resultado laboratorial. O sistema atual de notificação (Sistema de Informação de Agravos e Notificação — Sinan, Sistema de Informação da Vigilância Epidemiológica — Sivep) gera retrabalho e não facilita a tarefa para os profissionais. Além de emprestar tempo da atividade assistencial, muitas vezes a notificação não é realizada ou é inoportuna. Precisamos de um sistema que transmita automaticamente dados de registro de atendimento e resultados laboratoriais, filtrados por algoritmos específicos para o sistema de vigilância, que deve estar encarregado de limpar a base de dados, analisar e adotar as providências para controle de doenças e comunicação. Com a universalização da conectividade por telefonia celular, é fundamental que haja também a possibilidade de notificação por esta via, aumentando a sensibilidade e a oportunidade.
5. Revisão de métodos e processos de trabalho de vigilância e monitoramento de cada um dos temas de interesse incluídos na lista de notificação obrigatória e outros considerados relevantes. Cada doença e agravo tem dinâmica, característica e significado próprios. Entretanto, o modelo predominante de notificação universal passiva é aplicado atualmente a quase todos. Os processos de trabalho, as tecnologias e instrumentos devem ser revistos, de forma a contemplar as peculiaridades e aumentar a coerência com os objetivos estratégicos para cada doença e seu contexto (erradicação, eliminação, controle, redução de casos graves, sequelas e óbitos, contenção, monitoramento, redução de consequências sociais e econômicas). Há experiências com outros modelos; para vírus respiratórios, funciona um sistema híbrido, sentinela para síndrome gripal e universal para casos graves e óbitos, com fluxo hierarquizado para testes laboratoriais. As paralisias flácidas agudas são um exemplo de abordagem sindrômica sentinela para a detecção de vírus neurotrópicos, particularmente o poliovírus. Para vários agravos e doenças, a informação de casos individuais não é essencial para a detecção, o monitoramento e a adoção de medidas. O monitoramento de resistência a antimicrobianos pode ter um modelo que seja adequado ao serviço ou à localidade, outra lógica para a agregação de dados nacionais e ainda um desenho específico para determinados agentes multirresistentes.
6. Incorporação da vigilância baseada em eventos em todo o território nacional. O trabalho de rotina da vigilância epidemiológica pode ser mais oportuno e efetivo se agregado a um sistema de monitoramento de informações sobre possíveis eventos de interesse, detectados por meio de diversas fontes, como:
  - notícias e mídia (relatos de surtos ou incidentes publicados em jornais, *sites* de notícias, redes sociais e outras mídias);
  - observações de profissionais de saúde (relatos que podem sugerir doenças ou condições de saúde incomuns ou mudanças no padrão de ocorrência);
  - fontes comunitárias e de organizações (informações coletadas diretamente de comunidades locais ou de informantes chave, como agentes comunitários de saúde, que podem ter conhecimento de eventos de saúde antes que eles sejam formalmente registrados; comunicações de escolas ou profissionais de educação, assistência social, organizações religiosas, esportivas e outras que interagem com a população).
  O principal objetivo da vigilância baseada em eventos é detectar oportunamente possíveis ameaças à saúde pública, investigá-las e desencadear respostas imediatas. Isso é especialmente importante para o controle de surtos de doenças infecciosas, desastres ambientais, emergências químicas, biológicas, radioativas e outras ameaças.
7. Vigilância de doenças baseada em abordagem sindrômica. A primeira informação que surge sobre uma doença, tanto na clínica como na vigilância, é sobre sinais e sintomas dos pacientes. Pode não ser possível afirmar um diagnóstico etiológico (no caso das doenças infecciosas, como o tratado aqui), mas sim caracterizar as manifestações como síndromes. Isso permite escolher os passos seguintes para o diagnóstico, que frequentemente depende de exames laboratoriais, e adotar as primeiras medidas iniciais rapidamente, sem aguardar a confirmação do diagnóstico. A vigilância sindrômica inclui a detecção e o monitoramento de síndromes comuns, como doenças respiratórias (síndrome gripal, síndrome respiratória aguda grave), distúrbios gastrointestinais (diarreia, vômito), neurológicos (convulsão, alteração da consciência, dor de cabeça, confusão mental), além de sinais indicativos de doenças transmissíveis, como sangramento, icterícia, exantema. Tais informações podem vir de atendimentos em unidades de saúde (ou da busca em registros e prontuários, no caso de monitoramento), de núcleos de vigilância hospitalar ou mesmo de centrais de regulação e atendimento de emergências (SAMU, 190, 193). A avaliação contínua de prescrições de medicamentos e sistemas de resultados laboratoriais também podem ser fontes relevantes. Ao analisar os dados sindrômicos, a vigilância pode identificar alterações nos padrões que indiquem um evento antes mesmo de resultados laboratoriais. A vigilância sindrômica complementa os métodos tradicionais, fornecendo alerta que pode antecipar investigações e ações de controle.
8. Algoritmos de pesquisa laboratorial orientados pela vigilância sindrômica, com detalhamento genético e tecnologias para a identificação de patógenos emergentes. Durante a emergência, tivemos um intenso trabalho dos laboratórios centrais de saúde pública, de laboratórios de referência públicos, instituições de ensino e pesquisa e laboratórios privados. Essa foi a retaguarda para a confirmação de diagnósticos individuais e permitiu um monitoramento razoável da circulação de vírus. O acesso e a cobertura variaram muito e a oportunidade não foi das melhores. Foi preciso fazer atualização tecnológica em plena emergência. Em outras epidemias sempre há problemas semelhantes, com demora para aquisição de insumos, sobrecarga e longos tempos para resposta. A vigilância precisa de uma estrutura laboratorial organizada, com planos de acionamento, expansão de capacidade e fluxos que permitam racionalidade no uso dos recursos, rapidez e sensibilidade para a detecção de agentes de importância epidemiológica. Para que uma abordagem sindrômica seja possível, os algoritmos de testagem devem seguir a mesma lógica, com complexidade tecnológica crescente que chegue até a identificação de patógenos por metagenômica e outros métodos sofisticados, de modo que resultados finais negativos para agentes infecciosos sejam raros.
9. Acesso a diagnóstico: laboratório, autotestes (aplicativos de leitura e notificação); ainda que tecnologias para diagnóstico *in vitro* sejam organizadas para a lógica da vigilância, é importante garantir o direito de acesso individual a diagnóstico oportuno e confiável. Isso deve incluir a oferta de autotestes, em muitos casos, dando maior autonomia às pessoas, testes rápidos no local de atendimento e testes laboratoriais. É absolutamente viável formular métodos para a captação de informações adequados a cada uma das situações, o que pode aumentar muito a representatividade da vigilância.
10. Vacinas para controle de doenças e proteção individual: incorporação e caminhos para melhorar a cobertura. No Brasil é orgulho a existência do PNI, o mais amplo programa público desse tipo no planeta. Entretanto, persistem e renovam-se os desafios. Mencionada frequentemente como a mais efetiva entre as ações setoriais de saúde pública, a vacinação tem crescido em escopo, com a incorporação de novos imunizantes, de população alvo, que inclui todas as faixas etárias, de alcance geográfico e de complexidade. É preciso fortalecer a base operacional, começando pela atenção primária. Não basta uma sala onde haja armazenamento, aplicação, orientação, registro e trabalho gerencial. Da mesma forma, a equipe não deve se restringir a atividades operacionais. Para melhorar as coberturas e atingir os melhores resultados possíveis, é importante qualificar os processos em cada unidade ou equipe da Estratégia Saúde da Família, como planejamento, comunicação e diálogo com a comunidade, orientação individual, monitoramento de coberturas, identificação e busca de faltosos, redução de perdas de vacinas e conexão com outros programas, serviços e instituições. Isso exige gente preparada e estrutura. Duas frentes merecem ser sublinhadas, sem que tais menções signifiquem subestimar o relevante trabalho em curso. A incorporação de novas vacinas ao calendário oficial é resultado de várias etapas. A primeira é o conhecimento sobre a existência do produto, seja por monitoramento ativo, seja pela demanda de especialistas ou de empresas. O acesso ao produto depende de aprovação da Agência Nacional de Vigilância Sanitária — Anvisa (exceto em situações previstas em lei, de compra por organismos internacionais); e incorporação via Comissão Nacional de Incorporação de Tecnologias (CONITEC), com base em avaliação, que envolve características tecnológicas, necessidade, contexto epidemiológico, adequação ao SUS, manifestação da sociedade e custo. Durante a emergência por COVID-19, a agilidade, competência e transparência da Anvisa e do Ministério nesses campos foram impressionantes. Esse desempenho resultou de rearranjos emergenciais que não se sustentam em situação de rotina. É fundamental resgatar aspectos positivos daquele período que possam se tornar permanentes. A segunda etapa é o investimento em pesquisa, inovação e desenvolvimento na área de imunizantes. Isso não diz respeito apenas a financiamento, mas aos arranjos de organização que podem permitir passos mais rápidos tanto em tecnologias próprias como na transferência delas. É importante criar conexões sólidas entre as instituições produtoras e as de ensino e pesquisa. Não há hoje um caminho claro para que alguém se especialize e siga sua formação no tema; há pessoas que aprendem ao longo de carreiras nas indústrias e outras que buscam itinerários desenhados individualmente em suas trajetórias acadêmicas. As articulações para a produção das vacinas por Farmanguinhos e pelo Instituto Butantan certamente acrescentaram luz ao assunto. O Brasil tem experiência em políticas para a autossuficiência, que devem ser ampliadas e multiplicadas, com ênfase no necessário desenvolvimento da capacidade humana.
11. Inovação e produção de métodos para diagnóstico, prevenção, medicamentos e outras tecnologias. A organização da vigilância na esfera federal deve estar profissionalizada para uma atuação robusta em relação a esses temas, o que envolve:
  - prospecção do horizonte tecnológico;
  - avaliação e incorporação de tecnologias;
  - assuntos regulatórios, em estreita cooperação com a Anvisa;
  - acordos de cooperação;
  - financiamento, captação de recursos, políticas de investimento e prioridades;
  - proteção da propriedade intelectual e seus limites, dados pelas necessidades de saúde pública;
  - criação e desenvolvimento de programas de pesquisa e de formação.
  Não significa que laboratórios públicos não devam interagir, cooperar ou negociar com organizações de outros países, como aconteceu com as vacinas contra COVID-19, mas que o Governo Federal deve ter política para tanto e ter protagonismo, exercendo seu papel em negociação, fomento, celebração de acordos bi ou multilaterais e diplomacia, em seu sentido mais amplo, o que inclui o campo do direito internacional e a atuação com as organizações. Além dos interesses diretos do país, também deve ser lembrado seu papel na solidariedade entre os povos e na cooperação no âmbito do Sul Global.
12. Integração com áreas dedicadas ao estudo do clima, ação sobre o meio ambiente (natural e edificado) e a saúde animal. Se, há alguns anos, especulava-se sobre a possibilidade de que as mudanças climáticas produzissem efeitos sobre a saúde, hoje as consequências já são flagrantes, tanto por eventos extremos (inundações, calor extremo, seca) como pelo aumento da incidência de doenças, entre as quais são mencionadas frequentemente as de transmissão vetorial, que se expandem para latitudes e altitudes onde não havia transmissão. Cerca de 70% das doenças transmissíveis que emergiram nos últimos cem anos são zoonoses. Das sete emergências de saúde pública de importância internacional declaradas pela Organização Mundial da Saúde desde 2005, seis foram zoonoses e a sétima, motivada por aumento de casos de poliomielite, em 2014, tem importante determinação ambiental. Entre os exemplos, merecem menção os vírus respiratórios — principalmente o influenza — e a resistência a antimicrobianos. São problemas que só podem ser enfrentados com ações coordenadas que envolvam saúde ambiental, humana e animal. A expressão "One Health", utilizada aqui como "Saúde Única" ou "Uma só Saúde", tenta traduzir a preocupação de que sejam enfatizadas tais ligações, conhecidas há mais de cem anos. Há polêmicas que envolvem essas designações, mas não serão tratadas neste texto.
  Na prática, isso significa que: as organizações com responsabilidade sobre a saúde humana, de animais (de produção, silvestres ou de companhia), produção agrícola e meio ambiente devem planejar e agir de forma cooperativa, com intercâmbio de informações e planejamento conjunto. Trata-se de um objetivo ainda distante, pois elas têm missões, compromissos institucionais e formas de intervenção bastante diferentes.
  - A vigilância em saúde precisa expandir seu espectro de disciplinas e conexões, aproximando-se de entes ainda distantes, como os serviços de meteorologia, oceanografia, ecologia, órgãos de estudos e controle de poluição ambiental, proteção da biodiversidade e energia. É importante entender como as mudanças no ambiente acontecem, como a perda de biodiversidade e a degradação dos ecossistemas, e o que pode vir a influenciar a emergência de novos patógenos e a disseminação de doenças.
  - É preciso construir a capacidade de análise integrada. Influenza aviária e febre amarela são exemplos de que não pode haver compartimentalização das informações de interesse epidemiológico, sobretudo quando se considera a riqueza da fauna silvestre brasileira e a condição do país como grande criador de animais de produção.
13. Incorporação dos conhecimentos produzidos no campo das ciências sociais como subsídio para análise, planejamento, investigação e controle de doenças e agravos. As áreas de conhecimento envolvidas habitualmente no controle de doenças são insuficientes para enfrentar a complexidade do processo de determinação e adotar estratégias de controle efetivas. É importante compreender a dinâmica da população, vulnerabilidades, comportamentos, relações, cultura, crenças, saberes e desejos. Áreas outrora distantes são essenciais para o aperfeiçoamento da vigilância em saúde, como História, Economia, Pedagogia, Direito e Antropologia. A Epidemiologia, há algumas décadas, iniciou essa aproximação, entretanto ela não aconteceu de forma significativa nas instituições e serviços de saúde. Essa ampliação tem importância tanto para estudo, pesquisa e compreensão da dinâmica da população e das doenças como para a formulação das ações de prevenção, vigilância e resposta.
14. Atuação conjunta com assistência social, trabalho, previdência, finanças, educação, cultura e abastecimento. Algumas doenças e agravos são muito frequentes em certos segmentos da população e raros em outros, como os que decorrem da pobreza e concorrem para sua perpetuação, denominados doenças de determinação social, conforme atual programa prioritário do Governo Federal^2^. Muitos programas de controle dessas doenças incorporaram a prática da atuação integrada. A experiência acumulada com a AIDS e mais recentemente os programas de transferência de renda, já documentada em diversas publicações^13,14^, demonstra o quanto se pode ganhar em efetividade. Já no início da pandemia de COVID-19, ficou evidente o despreparo de vários setores para agir perante uma crise daquele porte. O confinamento de pessoas de baixa renda e trabalhadores informais em uma situação de emergência só é possível com suporte social. Isto vale também para o necessário isolamento de casos e comunicantes, que devem ficar afastados do trabalho e do convívio durante o período de transmissão. Estudantes só podem ficar em atividade remota se houver infraestrutura, planejamento e recursos para tanto. São numerosos os exemplos de ações intersetoriais para apoiar as intervenções para a contenção do vírus, dar suporte às pessoas e organizar o funcionamento da economia em situações excepcionais. É imprescindível um planejamento com a participação dos atores potencialmente envolvidos.
15. Trabalho integrado com a vigilância sanitária na prevenção e controle de doenças. Durante a emergência por COVID-19, tornou-se evidente para boa parte da população que a desconhecia o papel da vigilância sanitária na avaliação de imunobiológicos, medicamentos e métodos para diagnóstico usados na vigilância em saúde. A integração é uma necessidade quase óbvia para enfrentar as doenças de transmissão hídrica e alimentar, as infecções relacionadas à assistência à saúde, as exposições ambientais ou ocupacionais a patógenos, a resistência a antimicrobianos e aos inseticidas usados em controle de vetores, as doenças e agravos relacionados ao uso de tecidos, órgãos ou células, com a necessidade de regras para ventilação de ambientes coletivos e uso de máscaras. As vigilâncias, em conjunto, podem apontar para as conexões entre situações de risco e as doenças. Cabe à vigilância sanitária criar regras e zelar por seu cumprimento e à vigilância em saúde monitorar as ocorrências e a efetividade das regras implantadas, além de agir para o controle.
16. Ampliação do uso de técnicas de monitoramento ambiental para a detecção de doenças e agravos. São velhas conhecidas, mas pouco difundidas, as práticas de monitoramento de cursos de água e dejetos para vigiar a circulação de patógenos, como o vibrião colérico e o vírus da poliomielite. Técnicas laboratoriais mais sofisticadas permitem ampliar o espectro, de forma a detectar precocemente agentes e estimar a localização e intensidade de circulação, o que pode ser incorporado às práticas rotineiras de saúde coletiva como fonte rica de informações e subsídio para enfrentar doenças transmissíveis. Da mesma forma, a vigilância entomológica merece investimentos e a inclusão de novas tecnologias, várias já disponíveis, mas que não têm ultrapassado os limites de instituições de pesquisa.
17. Articulação com a sociedade para a detecção, resposta e suporte social. Existem organizações que há anos desenvolvem relevantes trabalhos no campo das doenças transmissíveis, principalmente as de longa duração, como hanseníase, tuberculose, AIDS, hepatites e doença de Chagas. Preocupam-se com o controle, mas principalmente com cuidado, tratamento e direitos dos portadores. Há alguma formulação teórica, experiências isoladas e outras efêmeras, sob diversas denominações, como vigilância civil, vigilância participativa, cidadã, comunitária, entre outras, com atuação mais ampla. O próprio Conselho Nacional de Saúde (CNS) formulou em 2018 uma resolução que trata da Política Nacional de Vigilância em Saúde^9^. Outrossim, a emergência por COVID-19 pôs à mostra o protagonismo de organizações da sociedade civil que atuaram em diversas frentes, incluindo comunicação, testagem, suporte econômico a pessoas vulnerabilizadas, fornecimento de alimentos, apoio ao isolamento. Iniciativas amplas como a Frente pela Vida, articulada ao CNS, o Todos pelas Vacinas, entre muitas, passaram a ter grande relevância naquele contexto. Mesmo setores empresariais, que habitualmente não têm grande interação com o setor saúde, juntaram-se a especialistas de saúde pública e constituíram uma entidade para captar recursos e apoiar a resposta à emergência: a iniciativa Todos pela Saúde, que deu origem a um instituto de mesmo nome.
18. Integração de setores e engajamento comunitário na gestão de emergências de saúde pública. A preparação e a resposta à pandemia devem ser analisadas para aprimorar a atuação em outros eventos futuros. A integração da vigilância com a atenção primária à saúde e com uma rede de laboratórios suficientemente equipada ofereceria informações oportunas para o contexto de emergência, compondo um eixo transversal da estrutura de gestão de riscos de desastres e emergências em saúde^15^. Áreas como segurança, educação e assistência social deveriam ser incluídas na formulação de planos de prevenção, preparação e resposta, bem como a sociedade civil organizada, cuja participação é essencial para promover o engajamento comunitário nas práticas preventivas e adesão às medidas de saúde pública, disseminar informações baseadas em dados e evidências científicas e ajustar as intervenções à realidade do território.
19. A incorporação de tecnologias inovadoras, com ampliação da capacidade dos sistemas de informação. Mais do que sistemas dedicados a registros específicos como doenças e agravos de notificação, é cada vez mais necessário trabalhar com as informações de grandes bases de dados, tanto da saúde como de outros setores (desenvolvimento social, previdência, trabalho, educação, segurança, trânsito, comércio, produção, clima etc.). Além de técnicas convencionais que já estão em uso, há ferramentas avançadas que a ciência de dados e a inteligência artificial podem oferecer para análises de grande volume de dados em tempo real, a fim de identificar padrões e tendências, permitindo intervenção imediata. Alguns trabalhos colaborativos têm mostrado o potencial dessas tecnologias, como o monitoramento de doenças relacionadas a vírus respiratórios, a modelagem estatística para projeções epidemiológicas (*nowcasting, forecasting*) e o cruzamento de bases de dados de doenças com as de programas sociais. É preciso ampliar as frentes de cooperação, como as que existem com o Centro de Integração de Dados e Conhecimentos para Saúde — CIDACS (Fiocruz-BA), o Info-gripe e o Info-dengue (Fiocruz, FGV) e o Laboratório Nacional de Computação Científica, e estabelecer capacidade própria de processamento de informações, com equipamentos, conectividade, organização, sistemas e equipe capacitada. A ampliação de capacidade deve resultar em disseminação ativa de informação oportuna e facilidade de acesso a dados epidemiológicos organizados para gestores, profissionais de saúde, pesquisadores, imprensa e a população em geral, conforme cada necessidade, em consonância com as leis de acesso à informação (LAI) e de proteção de dados (LGPD), evitando-se obstáculos burocráticos desnecessários.
20. Nova organização institucional, com quadro próprio, estabilidade e carreira. Toda a estrutura de vigilância em saúde na esfera federal hoje está contida na Secretaria de Vigilância em Saúde e Ambiente do Ministério da Saúde. Como parte da administração direta, há obstáculos para o melhor desempenho da área. Entre eles, vale mencionar a falta de autonomia orçamentária e administrativa, que dificulta a resposta oportuna em situações em que o tempo é um fator crítico, como nas emergências, e a fragilidade do quadro funcional. São poucos os servidores públicos estáveis no Ministério da Saúde que têm a formação e a experiência necessárias. Isso compromete a continuidade do trabalho e inviabiliza o necessário aperfeiçoamento contínuo em temas de muita complexidade.
21. Além desses aspectos, é importante olhar para a vigilância em saúde como uma atividade de Estado, e não de um governo específico. Embora possa haver prioridades circunstanciais ou políticas, que correspondem a programas de governo, trata-se de uma atividade contínua, cujo bom funcionamento repercute na segurança sanitária do país e, portanto, na redução de doenças e mortes evitáveis. Assim, é desejável que a direção da área seja protegida por mandato, com estabilidade para garantir que não haja interrupções em ações e programas quando há transições de governo ou disputas políticas. Não se pode admitir novamente a ausência completa de coordenação nacional, como aconteceu durante um período tão crítico como o vivido entre os anos de 2020 e 2022, quando o Ministério da Saúde navegava sem rumo, ao sabor de discursos e decisões do comando do governo que negavam os conhecimentos científicos e a gravidade da situação.

Como inscrito na Constituição Federal e na legislação infraconstitucional, a governança é compartilhada de forma federativa entre União, estados (representados por sua entidade, o Conselho Nacional de Secretários de Saúde — CONASS) e municípios (representados pelo Conselho Nacional de Secretarias Municipais de Saúde — CONASEMS), com instâncias definidas para discussão, negociação e decisão.

Além disso, é importante valorizar a participação social, via CNS, que tem até mesmo uma comissão específica para o tema, e da comunidade científica, por comissões técnicas dedicadas.

A vigilância em saúde no Brasil foi organizada de forma integrada como um sistema há meio século e passou a integrar o SUS quando este foi criado pela Constituição de 1988, capilarizando-se por todos os estados e municípios brasileiros. Neste período, avançamos muito, com sistemas organizados e rotinas de atuação, obtendo resultados expressivos na eliminação e no controle de diversas doenças. O modelo implantado passou por adaptações ao novo contexto institucional após a promulgação da Constituição Federal, mudanças de organização e modernização tecnológica, mantendo, entretanto, seus fundamentos conceituais e a lógica de funcionamento originais.

Essa modernização não foi suficiente para garantir uma vigilância em saúde eficaz, capaz de fazer frente a uma epidemia como a de COVID-19, em um período de grande adversidade política e fragilidade institucional.

Os desafios enfrentados na pandemia da COVID-19 incluíram a necessidade de uma readaptação nos modelos de vigilância, em face da incapacidade de uma ação enérgica no momento de uma emergência de saúde pública global. Infelizmente, o atual sistema de vigilância e assistência em saúde não se mostrou efetivo para detecção precoce e resposta.

A vigilância em saúde precisa ser resiliente e ajustável, não só para reagir, detectar e dar resposta, mas também para antecipar as crises de saúde, sendo capaz de se adaptar e se recuperar diante de desafios imprevistos e contínuos.

A experiência revelou o quão essencial é um sistema de saúde flexível, integrado e articulado com setores de governo e diversos atores da sociedade para uma resposta coordenada e eficaz.

Um dos pontos mais frágeis foi a falta de equidade nas ações de vigilância em saúde, com ações insuficientes para a proteção de diferentes segmentos, como comunidades de baixa renda, grupos minoritários, moradores de áreas rurais ou os que têm menos acesso a recursos, informações e cuidados na prevenção e controle de doenças, tendo como agravantes a desigualdade de acesso a tecnologias e a falta de letramento digital.

A experiência brasileira com a COVID-19 ofereceu valiosas lições para a construção de um sistema de saúde mais preparado para desafios presentes e futuros e, principalmente, para promover a equidade. Este artigo contribui com reflexões para o futuro da vigilância em saúde, que precisa urgentemente, como política pública essencial, revisitar e reestruturar conceitos, abordagens, tecnologias e sua organização institucional, com ênfase em aspectos fundamentais. Alguns destes foram citados aqui, mas cada um é merecedor de um capítulo específico para subsidiar reorientações do sistema e guiar a formulação de políticas e práticas.

## Footnotes

1 Na trajetória do SUS, a nomenclatura adotada pelo Conselho Nacional de Saúde e pelo Ministério da Saúde passou a tratar como Vigilância em Saúde a organização institucional da Vigilância Epidemiológica.

2 Instituído pelo Decreto n° 11.908, de 6 de fevereiro de 2024, o Programa Brasil Saudável: unir para cuidar é um desdobramento das ações do Comitê Interministerial para a Eliminação da Tuberculose e de Outras Doenças Determinadas Socialmente. São mencionadas nominalmente: tuberculose, doença de Chagas, esquistossomose, filariose linfática, geo-helmintíases, malária, oncocercose, tracoma, HIV/AIDS, hepatites virais, hanseníase e HTLV.
